# Pleiotrophin-Neuregulin1 promote axon regeneration and sorting in conduit repair of critical nerve gap injuries

**DOI:** 10.21203/rs.3.rs-3429258/v1

**Published:** 2023-11-06

**Authors:** Xingjian Gu, Farial S. Rahman, G Bendale, B Tran, JF Miyata, A Hernandez, S Anand, Mario I. Romero-Ortega

**Affiliations:** 1 Department of Biomedical Engineering, University of Houston, Houston TX 77204; 2 Department of Biomedical Engineering, University of Arizona, Tucson AZ 85721

**Keywords:** neuregulin, nerve regeneration, Schwann cells, radial sorting, remyelination, growth factor

## Abstract

Significant challenges remain in the treatment of critical nerve gap injuries using artificial nerve conduits. We previously reported successful axon regeneration across a 40 mm nerve gap using a biosynthetic nerve implant (BNI) with multi-luminal synergistic growth factor release. However, axon sorting, remyelination, and functional recovery were limited. Neuregulin1 (NRG1) plays a significant role in regulating the proliferation and differentiation of Schwann cells (SCs) during development and after injury. We hypothesize that the release of NRG1 type III combined with pleiotrophin (PTN) in the BNI will enhance axon growth, remyelination, and function of regenerated nerves across a critical gap. A rabbit 40 mm peroneal gap injury model was used to investigate the therapeutic efficacy of BNIs containing either NRG1, PTN, or PTN+NRG1 growth factor release. We found that NRG1 treatment doubled the number of regenerated axons (1276±895) compared to empty controls (633±666) and PTN tripled this number (2270±989). NRG1 also significantly increased the number of SOX10^+^ Schwann cells in mid-conduit (20.42%±11.78%) and reduced the number of abnormal Remak axon bundles. The combination of PTN+NRG1 increased axon diameter (1.70±1.06) vs control (1.21±0.77) (p<0.01), with 15.35% of axons above 3 μm, comparable to autograft. However, the total number of remyelinated axons was not increased by the added NRG1 release, which correlated with absence of axonal NRG1 type III expression in the regenerated axons. Electrophysiological evaluation showed higher muscle force recruitment (23.8±16.0 mN vs 17.4±1.4 mN) and maximum evoked compound motor action potential (353 μV vs 37 μV) in PTN-NRG1 group versus control, which correlated with the improvement in the toe spread recovery observed in PTN-NRG1 treated animals (0.64±0.02) vs control (0.50±0.01). These results revealed the need of a combination of pro-regenerative and remyelinating growth factor combination therapy for the repair of critical nerve gaps.

## Introduction

Peripheral nerve injury results in partial or complete loss of sensory, motor, and autonomic functions, with over 200,000 new cases and $150 billion in healthcare costs annually in the United States ^1^. For nerve injuries with small gap length (<8 mm), a tensionless epineural coaptation is performed to reapproximate the proximal and distal nerve stumps with micro-sutures ^2^. For nerve gaps up to 20 mm, several artificial nerve conduits made of natural or synthetic polymers have been approved by FDA for bridging the injured nerves. For critical nerve gaps (>30 mm), autologous nerve transplantation (autograft) remains the clinical gold standard, despite being inevitably associated with multiple surgeries and donor site morbidities ^3^. Acellular nerve allografts have been proposed as an alternative to autograft, but they show limited regenerative potential in nerve gaps of >30 mm ^4,5^. When the gap length exceeds 40 mm, functional recovery is limited, characterized by reduced axon number in the distal end, insufficient remyelination, and muscle atrophy, even after autograft repair ^2^. To develop nerve conduits with improved therapeutic outcome for critical nerve gap injury, different strategies have been proposed, including incorporation of intraluminal fillers that resemble the natural scaffold provided by the Bands of Büngner, adding Schwann cells (SCs), using electrical stimulation, and incorporating the delivery of exogenous growth factors such as glial-derived neurotrophic factor (GDNF) ^3,6–8^. While these strategies offer some advantage over empty conduits, their regenerative potential in in critical long gaps has been limited.

In our previous work, we used BNIs with sustained release of GDNF and pleiotrophin (PTN) to synergistically entice motor and sensory axon to regrow. This molecular combination induced successful axon regeneration across a 40 mm gap of the rabbit common peroneal nerve ^9^. However, distal to the conduit we observed almost exclusively, bundles of unmyelinated mixed-caliber axons in uncharacteristic large numbers forming abnormally large Remak bundles. This observation revealed the inability of pro-regenerative SCs in long gap injuries to interact with regenerative axons, stalling in stage 1 of the radially axon sorting process ^10^. Regenerated axons are normally ensheathed by Remak SCs in groups of 10–20, and those above 1μm in diameter are normally individually sorted out by differentiated SCs and subsequently myelinated, where the myelin thickness is proportional to the expression level of membrane-bound NRG1-III ^11,12^, leaving bundles of 5–9 small axons ensheathed by non-myelinating Schwann cells ^12,13^. This axonal radial sorting process is dependent on neuronal membrane expression of neuregulin-1 type III (NRG1; also known as sensory and motor neuron-derived factor, SMDF), which is strongly and transiently up-regulated in the distal nerve stump after nerve injury ^14–16^, and processed into a myelinationin-ducing variant by the protease BACE1 (β-site amyloid precursor protein–cleaving enzyme1) ^17^. The signaling domain of NRG1 binds to ErbB2/3 receptors on pro-regenerative Schwann cells and influence their migration and differentiation into a pro-myelinating phenotype ^18–20^. Both soluble and transmembrane isoforms of NRG1, activate the ErbB pathway and play a critical role in myelination ^21,22^. The soluble form is particularly important for SCs survival, migration, dedifferentiation, and myelination ^16,23–25^. Axonal radial sorting relies on the deposition of extracellular matrix molecules, and the establishment of polarity in premature Schwann cells, through activation of the Akt-Gsk3β axis, and ILK, FAK, RhoGTPases, Merlin/Nf2 pathways. This was supported by the observation that inactivation of Gab1 (Grb2 associated binder 1), a scaffolding molecule downstream of NRG1-Erk/Akt pathway in SCs, generates abnormal Remak bundles containing unsorted large caliber axons ^26^. In fact, mice lacking NRG1 expression, also showed abnormally large Remak bundles where axons of different sizes remained unsorted ^27^, highlighting the critical role of this axon derived molecule in axon sorting and remyelination.

Since SCs upregulate and secrete NRG1 immediately after nerve injury ^28^, we reason that the lack of axon sorting in BNIs with growth factor support might be explained by either a low number of SCs, or insufficient expression of NRG1 by these cells in long gap repairs, which require a longer time for regeneration and functional recovery. We hypothesized that adding in the lumen of the nerve conduits soluble NRG1 to activate immature SCs, and neural growth factors to mediate axonal regeneration, will enhance nerve regeneration, remyelination and functional recovery in long-gap nerve repairs. In this study, we combined polymeric microparticle encapsulation of soluble NRG1, added to activate immature Schwann cells and induce them to proceed with axon sorting and improve remyelination, and PTN, added to stimulate axonal regeneration ^29^, across a 40 mm rabbit common peroneal nerve gap injury. We found NRG1 and PTN both promoted axon regeneration. Furthermore, NRG1 increased the number of SOX10^+^ Schwann cells in the BNI conduits and improved the sorting of regenerated axons. Although the number of remyelinated axons was not significantly increased, behavioral studies confirmed the improvement of functional recovery in PTN-NRG1 treated animals. Together, our results support the use of a combination therapy including growth factor and pro-myelinating factor combination to induce axon regeneration, radial sorting, and functional recovery across a critical nerve gap repairs.

## Results

### Microparticle characterization and in vitro bioactivity assay

**Figure 2.**
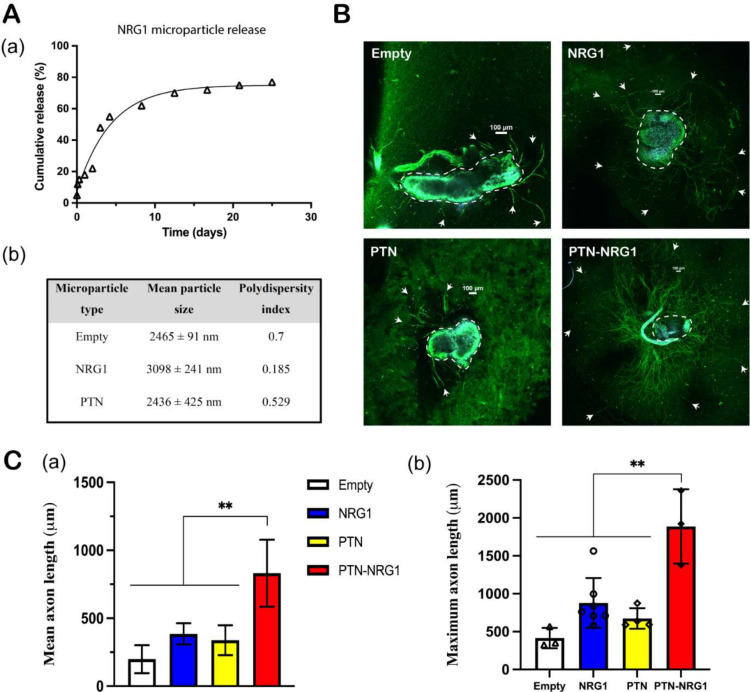
Microparticle release of NRG1. **A**, (a) 28-day NRG1 release profile; (b) Characterization of the PLGA microparticle size and polydispersity index. **B**, Representative confocal images of DRG explants. DRGs were immunostained for axon (β-tubulin, green) and cell nuclei (DAPI, blue). Neurites were highlighted by white arrows while cell bodies were highlighted in yellow dashed circles. Scale bar: 100 μm. **C**, Quantification of the mean and maximum neurite length of DRG explants cocultured with empty, NRG1, PTN, and PTN-NRG1 microspheres for one week. **, p<0.01.

As determined from zeta potential analyzer, the mean particle size of empty, NRG1 and PTN-NRG1 PLGA microparticles was 2.41±0.1μm, 3.09±0.2 μm, and 2.43±0.4 μm; respectively (Fig. 2A–a). The polydispersity index was 0.7, 0.185, and 0.529 for empty, NRG1 and PTN-NRG1microparticles, respectively (Fig. 2A–b). The *in vitro* release kinetics of NRG1 from the microparticles was determined by suspending 10 mg microspheres in 1 ml PBS at 37°C and collecting the solution at regular intervals spanning one month. The growth factor content in the release samples was quantified by ELISA. The PLGA microparticles provided a sustained release of protein over a period of twenty-six days, as shown in Fig. 2A. The *in vitro* release of PTN from PLGA microparticles was published in our previous study ^9^. Since NRG1 Type-III has not been previously applied *in vivo*, we used the dose of NRG1-β1 as reference for the subsequent *in vitro* bioactivity assay and *in vivo* animal study. Intramuscular application of 2 μl of 10 nM NRG1-β1 has been shown to improve Schwann cell proliferation and differentiation, and to promote regeneration in an end-to-side neurorrhaphy model ^30^. This concentration is comparable to that used for Schwann cell stimulation at the neuromuscular junction, and thus considered adequate for stimulating axon remyelination.

To assess whether the encapsulated growth factors were biologically active, dorsal root ganglia (DRG) explants were co-cultured with the growth factor loaded microparticles. Empty, NRG1, PTN, and PTN-NRG1 microparticles were mixed with neutralized collagen solution, added to the DRG culture, and incubated at 37°C in neurobasal-A media. The mean and maximum neurite length were quantified after co-culture for one week. As seen in Fig. 2B–C, neurite outgrowth was observed in all groups treated with growth factor loaded microparticles. While NRG1 (mean 384.5±78.11 μm, max 879±328.4 μm) and PTN (mean 338.1±110.4 μm, max 673.8±135.6 μm) alone stimulated the growth of axons compared with empty control (mean 197.9±103.1 μm, max 414.7±133.6 μm), the combination of PTN and NRG1 resulted in the highest mean (831.5±246.7 μm) and maximum length (1887±490.8 μm) of axon outgrowth. Since PTN and NRG1 showed a synergistic effect on axon growth, this combination was further evaluated *in vivo*.

### Gross anatomical analysis of regenerated nerves

**Figure 3.**
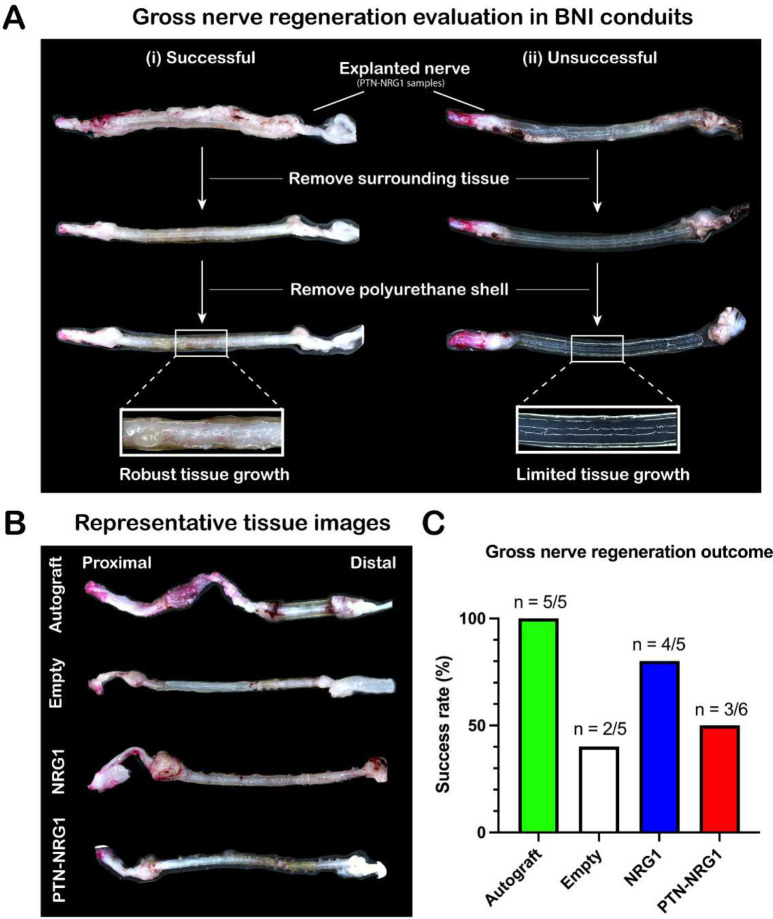
Nerve repair efficacy. Gross anatomical evaluation of tissue after removal from the conduits. **A**, Characteristic cases of successful (i) and unsuccessful (ii) gross nerve regeneration in the PTN-NRG1 treated group. **B**, Representative images of nerve tissue obtained from autograft, control, NRG1 and PTN-NRG1 groups. **C**, Quantification of the gross nerve regeneration outcome. The NRG1 group showed higher number of successfully regenerated samples than empty control and PTN-NRG1 groups when compared to the autograft controls.

The explanted nerves were inspected for tissue growth and bridging of the nerve stumps, to confirm the successful regeneration across the 40 mm gap (Fig. 3). There was observable fibrotic tissue growth covering all the BNI tubes, with apparent tissue growth in most of them. However, after removing the regenerated tissue from the polyurethane conduits, we found that only some of the nerves were fully regenerated across the gap with robust tissue growth and vascularization, as shown in (Fig. 3A, i). In some tubes, the agarose microchannels were intact with little to no tissue present in the regenerative scaffold (Fig. 3A, ii). To evaluate the gross nerve regeneration outcome, samples showing fully connected regenerative bridge with vascularized epineural layer (regenerated tissue between polyurethane shell and agarose hydrogel) were classified as success (Fig. 3B). Using this criterion, all animals in the autograft group (5/5), 40% of control (2/5), 80% of NRG1 (4/5) and 50% of PTN-NRG1 (3/6) animals showed successful regeneration across the 40 mm gap (Fig. 3C). Only samples with confirmed tissue growth in the conduits were included for subsequent analysis.

### PTN-NRG1 promotes axon regeneration across 40 mm critical gap

**Figure 4.**
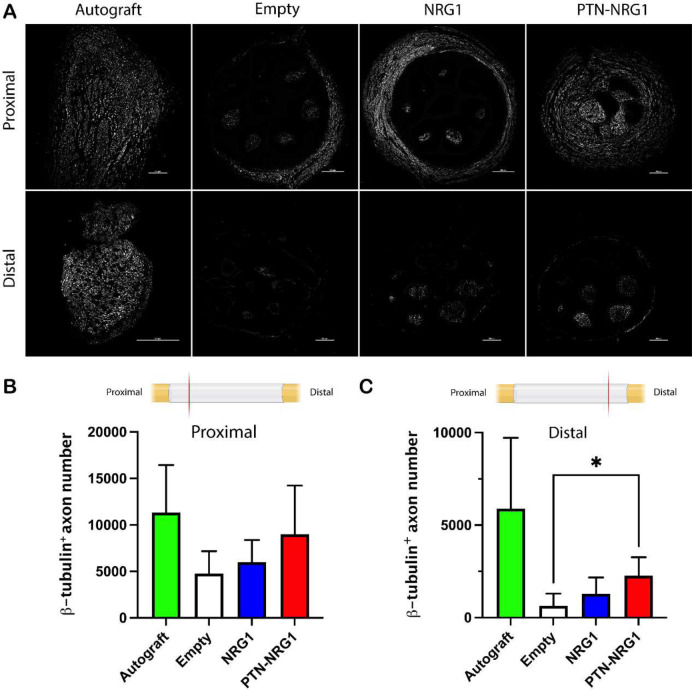
Axon regeneration into the distal nerve stump is limited. **A**, Representative confocal images of β-tubulin III labeled axons showed limited regeneration into the distal segments. **B-C**, Quantification of the β-tubulin^+^ axon in the proximal (**B**) and middle (**C**) segments showed enhanced axon number in the PTN-NRG1 treated group but reduced compared to autograft repair. Scale bar: 200 μm. *, p<0.05.

The proximal and middle sections of the nerves were evaluated histologically and labelled with β-tubulin III to quantify the number of axons regenerated across the 40 mm gap (Fig. 4). In the proximal segment, the BNI with no growth factor support showed 4767±2407 axons compared to those with NRG1 5990±2377, while PTN+NRG1 induced the growth of 9016±5221, comparable to those observed in autograft repair (11320±5127; Fig. 4B). However, the number of regenerated axons reaching the distal segment of the conduit reduced dramatically (Fig. 4C). In the empty group less than 15% (633±666) were observed distally. This percentage increased to 21% (1276±895) in the NRG1 and to 25% in the PTN-NRG1 (2270±989) groups, which was significantly larger compared to those without growth factor support, but remained lower than that with autograft repair 52% (5897±3822). The reduced axon number in BNI groups between the proximal and distal end as compared with autografts, could be attributed to insufficient molecular or cellular support in the center of the conduit, which in some degree also seemed to affect nerve regeneration in the autograft group.

### NRG1 release in the BNI is sufficient for proliferation and migration of SCs

**Figure 5.**
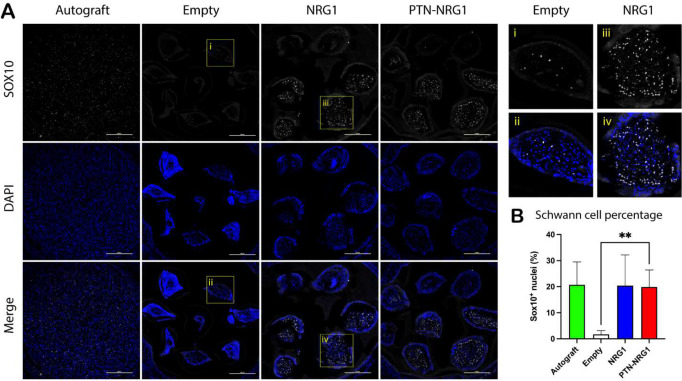
NRG1 increases SC cell number in mid nerve conduit. **A**, Representative confocal images showing SOX10^+^ Schwann cell nuclei (white) and DAPI stained cell nuclei (blue). Top right panel shows magnified view of highlighted areas. Scale bar: 200 μm. **B**, Quantification of the percentage of Schwann cell nuclei (SOX10/DAPI ratio) in the middle segment of the tissue. NRG1 dramatically increased Schwann cell number in the middle segment of BNI conduits. **, p<0.01.

Given the critical role of regenerative SCs for nerve repair, we were interested in learning how the growth factors affected these cells in the damaged nerve tissue. Cross-sections of explanted nerve tissue from the middle section of the BNI were immunolabelled with a SOX10 antibody to visualize the nuclear expression of this transcription factor in pro-regenerative SCs. As expected, SOX10^+^ cells were observed in the autograft repaired nerve tissue (Fig. 5A). Surprisingly, very few pro-regenerative SCs were present in the middle segment of BNIs with no growth factor release (1.69±1.44%). In contrast, NRG1 in the BNI conduit greatly increased the number of Schwann cells in the conduit (20.42±11.78% and 19.89±6.56% for NRG1 and PTN-NRG1 groups, respectively), to a level comparable to that observed in the autograft (20.73±8.79%) (Fig. 5B). The increase in SOX10^+^ SCs in the middle of the conduit is consistent with the known stimulatory effect of NRG1 on migration and proliferation of repair SCs.

### Radial axon sorting is increased by NRG1 release in BNI

Next, we evaluated if NRG1 release in the BNI stimulated radial axon sorting of regenerative SCs (Fig. 7). The number of axons wrapped inside Remak bundles were quantified, and those containing more than 14 axons were considered as evidence of abnormal radial sorting ^31^. In the autograft group we observed only 3 Remak cells with an average of 17.67±1.16 axons/bundle. This number was dramatically increased to 36 bundles in the negative control group with an average of 21.64±6.18 axons/bundle, indicating that the lack of SOX10^+^ cells in this group correlated with lack of radial axon sorting in repaired nerves. The number of Remak bundles, and axons per bundle were reduced in the NRG1 (10 bundles, 15.80±1.23) and the PTN-NRG1 (3 bundles, 16.33±1.53) to levels comparable to the autograft repaired group (Fig. 7B). Quantification of axon diameter revealed that the mean axon size in autograft were 1.76±1.33 μm, with smaller axons observed in the empty (1.21±0.77 μm) and NRG1 (1.08±0.58 μm) groups. Compared to empty BNI with average axons of 1.21±0.77 μm and only 3.55% of them larger than 3 μm, the release of NRG1 in the BNI did not alter axon diameter size distribution as it was similar compared to the negative control group with only 1.82% of them being larger than 3 μm. In contrast, those with NRG1-PTN showed significantly larger axons (1.70±1.06 μm) and 15.35% of them were larger than 3 μm, comparable to that of the autograft group (17.81%) (Fig. 7C–D). The increased axon diameter is consistent with previous report on the effect of sustained neurotrophic support in BNI critical gap regeneration ^9^.

### NRG1 promotes axon remyelination

**Figure 6.**
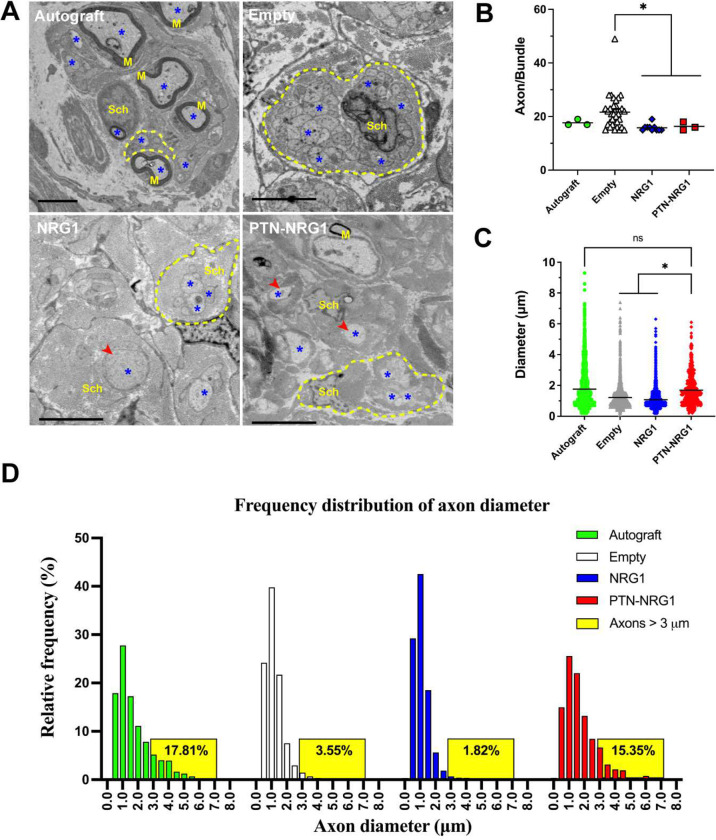
NRG1 promotes remyelination. **A**, Toluidine blue staining of the regenerated tissue Scale bar: 200 μm. **B**, TEM images of the myelinated axons in the distal end of regenerated nerves. Scale bar: 5 μm. **C**, Average number of remyelinated axons observed per view field. **D**, Diameter of the remyelinated axons. **E**, Analysis of g-ratios for remyelinated axons observed in each group. One-way ANOVA was used to compare means between groups. *, p<0.05; ****, p<0.0001; ns, p>0.05.

**Figure 7.**
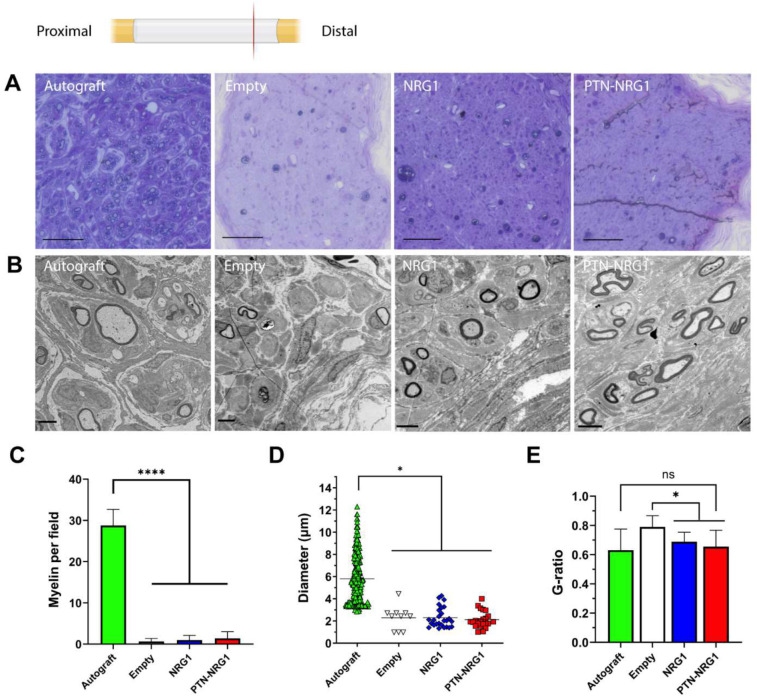
NRG1 stimulated axon sorting and PTN increased the regeneration of mid-size axons. **A**, Representative electron microscopy images showing axons within Remak bundles. Axons, myelin, Schwann cells are labeled by a star symbol, M, and Sch, respectively. Remak bundles are highlighted by yellow dashed circles. Red arrowheads indicate large unmyelinated axons sorted out of the Remak bundles. Scale bar: 5μm. **B**, NRG1 significantly reduced the number of abnormal axon bundles, indicating the positive effect on axon sorting. **C**, Quantification of the diameter of myelinated and unmyelinated axons present in the distal end of the regenerated nerves. **D**, Frequency distribution of the axon diameter. PTN-NRG1 treatment increased the number of axons above 3 μm in diameter, comparable to that of the autograft group. *, p<0.05; ns, p>0.05.

The explanted tissues were stained with toluidine blue to reveal the cellular morphology of regenerated nerves (Fig. 6A). As expected, the autograft repaired nerves showed successful remyelination of regenerated axons. However, all the BNI repaired nerves showed very limited number of remyelinated axons. To further analyze the effects of NRG1 on pro-regenerative SCs, the regenerated nerves at the distal end were imaged by transmission electron microscopy to visualize the cellular ultrastructure (Fig. 6B). The autograft group had significantly higher number of myelinated axons (28.75±3.92 per field) than the empty (0.64±0.74 per field), NRG1 (0.99±1.11 per field) and PTN-NRG1 (1.40±1.63 per field) groups (Fig. 6C). NRG1 did not increase the number of myelinated axons. Quantification of the axon diameter (Fig. 6D) showed that the myelinated axons in autograft group had an average diameter of 5.80±2.16 μm, while in the BNI groups the myelinated axons were of similar diameter: control (2.28±1.08 μm), NRG (2.30±0.90 μm) and PTN-NRG1 (2.12±0.76 μm). Quantification of myelin thickness (Fig. 6E) revealed that the autograft group had significantly lower g-ratio than the empty control (0.63±0.14 vs 0.79±0.08), suggesting that the absence of growth factors in the empty conduit caused insufficient axon remyelination after injury. The delivery of NRG1 by PLGA microparticles induced a reduction in g-ratios in the NRG1 (0.69±0.07) and the PTN-NRG1 (0.65±0.11) groups comparable to that in the autograft group.

### PTN-NRG1 induces higher CMAP amplitude and motor force recruitment

**Figure 8.**
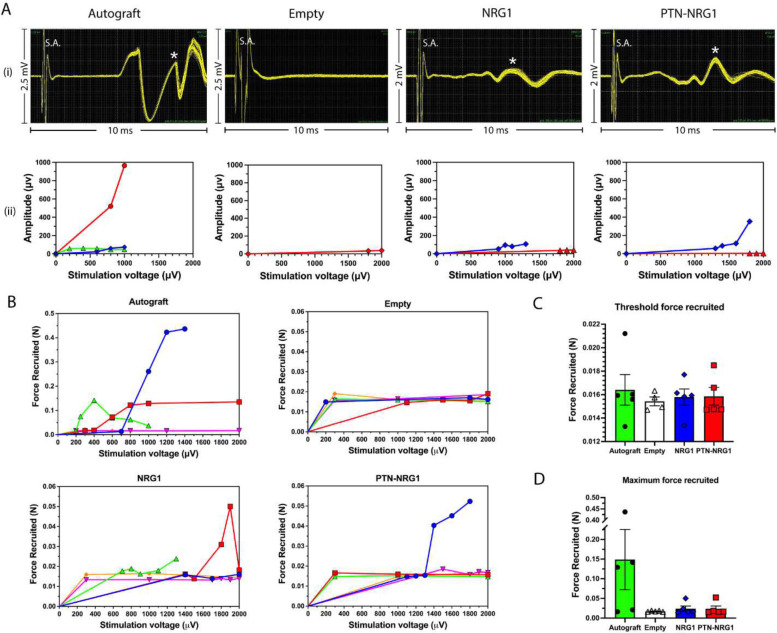
Limited recovery of motor function by growth factor loaded BNIs. Recording of compound muscle action potential (CMAP) and muscle force recruitment. **A**, (i) Representative CMAP waveforms for each group; (ii) CMAP amplitudes at different stimulation voltages. S.A. represents stimulation artifact. Star symbols indicate peaks selected for analysis. **B**, Muscle force recruited at different stimulation voltages. **C-D**, Quantification of threshold force recruited, and maximum force recruited.

To evaluate nerve conduction and muscle reinnervation, regenerated nerves were stimulated at the proximal stump and recorded at the tibialis anterior muscle for compound muscle action potential. Additionally, motor force recruited by the muscle was recorded using a force transducer. For compound muscle action potential (Fig. 8A), the autograft group had the highest maximum CMAP amplitude (962 μV), followed by the PTN-NRG1 group (353 μV) and NRG1 group (109 μV), with the lowest in the empty group (37 μV). Similar results were observed for muscle force recruitment (Fig. 8B–D). Growth factor treatment resulted in greater muscle force recruited at the activation thresholds (Fig. 8C). The maximum muscle force recruited (Fig. 8D) was highest in the autograft (148.9±171.1 mN), followed by the NRG1 (24.1±15.0 mN), PTN-NRG1 (23.8±16.0 mN) and the lowest in the empty group (17.4±1.4 mN). These results suggest that the axons in the BNI groups were correctly guided to the target muscle, and that PTN-NRG was more effective in mediating muscle contraction, also showed that this combination of growth factors are insufficient to match the regenerative effect of the autograft.

### Muscle atrophy despite method of nerve repair

**Figure 9.**
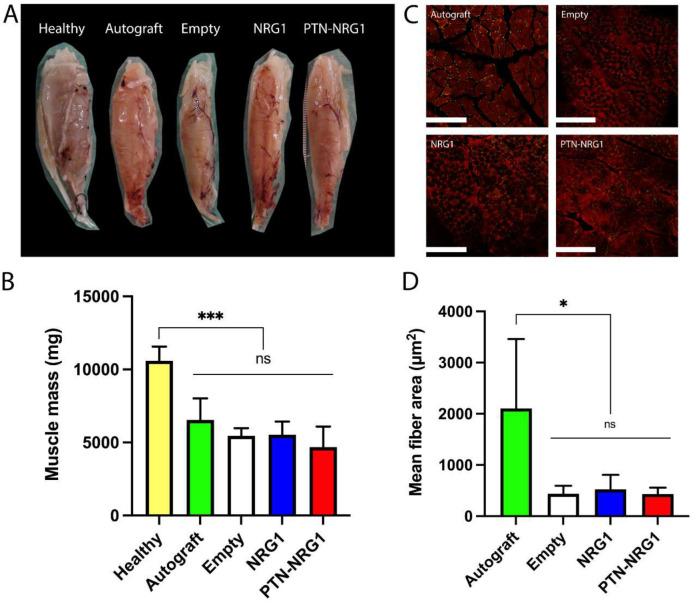
No effect of nerve repair on tibialis muscle atrophy. **A**, Representative gross tissue images of the tibialis anterior muscles obtained after perfusion. **B**, All groups demonstrated significant muscle atrophy at the end of 28-week recovery period. **C**, Representative images of cross sections of muscle tissue, demonstrating the difference in muscle fiber area between autograft and BNI groups that is quantified in **D**. *, p<0.05; ***, p<0.001.

To evaluate the degree of muscle re-innervation after regeneration, we measured the wet muscle mass of the tibialis anterior muscle collected after perfusion. Compared with healthy muscles (10.59±0.98 g), all groups had a significant loss of muscle mass (p<0.0001) (Fig. 9A). The autograft group lost 38% of its muscle (6.54±1.48 mg), and this was not different from those with BNI repair: empty (5.46±5.23 g), NRG1 (5.54±0.89 g), and PTN-NRG1 (5.19±0.74 g) (Fig. 9B). This indicates that despite the robust axon regeneration and remyelination in autografts, significant muscle atrophy occurs due to nerve injury and delayed re-innervation. We further quantified the muscle fiber area from images taken for the neuromuscular junctions (Fig. 9C). The autograft group had the largest area of muscle fibers (2106±1355 μm^2^), indicating robust re-innervation, as compared with the BNI groups (Fig. 9D). While similar in number, the NRG1 (525±283 μm^2^), had slightly higher fiber areas as compared with the empty (437±159 μm^2^) and the PTN-NRG1 groups (436±121 μm^2^).

### PTN-NRG1 promotes early, sustained functional recovery

**Figure 10.**
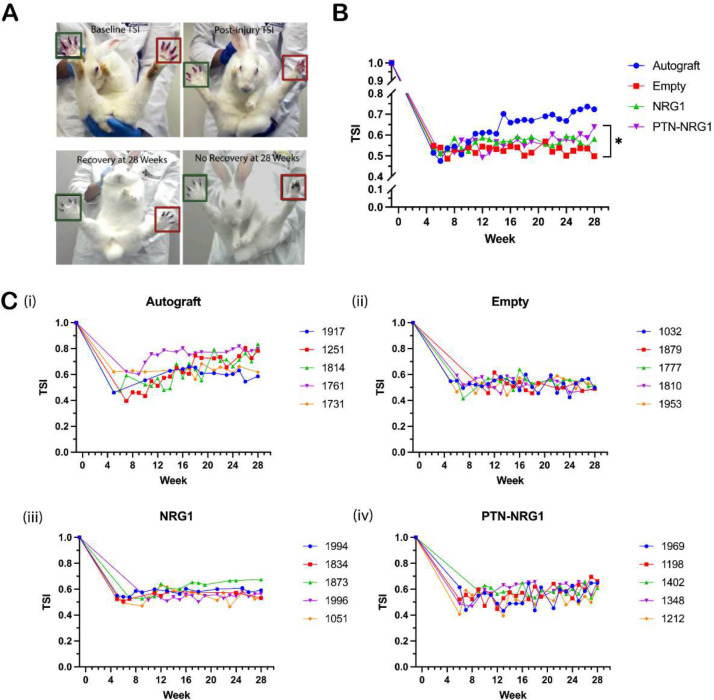
Moderate improvement of motor function by PTN-NRG1 BNIs. **A,** Illustrative images of toe-spread index (TSI) measurement, and examples of baseline, injury, recovery, and no recovery toe-spread. Green and red boxes correspond to uninjured and injured side, respectively. **B,** Mean TSI for different groups over the 28-week recovery period. PTN-NRG1 combination significantly increased TSI, compared with the empty control group. **C,** TSI value of individual animal for different groups. The 4-digit numbers are random codes generated for double-blind analysis, each representing an individual animal. Sample size n = 5 for all treatment groups. *, p<0.05.

Over the 28 weeks, toe-spread index (TSI) was measured by calculating the distance between the first and the last toe during the startle response of the animals (Fig. 10A). At five weeks post-injury, all animals had functional deficits compared with baseline, although some animals in the autograft exhibited signs of recovery (Fig. 10B). At the end of 28 weeks, the autograft group had an average TSI of 0.72±0.11, significantly higher than that of the empty (0.50±0.01) and NRG1 group (0.58±0.06). Growth factor treatment using the PTN-NRG1 combination resulted in a moderate (0.64±0.02) but statistically significant improvement of TSI compare to the empty group (p<0.05). These results highlight the significance of axon sorting and target reinnervation in addition to axon growth during nerve regeneration, indicating that albeit moderate, effects of NRG1 on migration and proliferation of SCs may have contributed to a slightly superior functional recovery.

### Axonal NRG1 expression was downregulated in 40 mm nerve gap injury

The above results showed that NRG1 delivery successfully induced Schwann cell migration and proliferation into the BNI conduits, however, axon remyelination was not significantly improved by these Schwann cells. Because the myelination process of peripheral axons is regulated by both Schwann cells and axon-derived signals, we investigated the expression of membrane-bound NRG1 type III on the regenerated axons.

**Figure 11.**
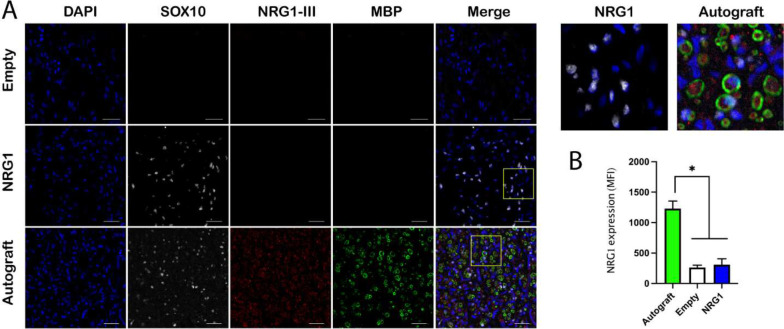
Axonal NRG1 type III is down-regulated in critical nerve injuries. **A,** NRG1 treated animals showed a significant increase of SOX10 positive Schwann cells in the conduit compared with the empty group, but remyelination was limited. In contrast, the axons in the autograft group showed significantly higher expression of axonal NRG1 type III and remyelination than the empty and NRG1 groups. Top right panel shows magnified view of highlighted areas. Scale bar, 20 μm. **B,** Quantification of the axonal NRG1 type III expression in all groups. Autograft group showed significantly higher MFI value than those of empty and NRG1 groups. *, p<0.05.

The axonal NRG1 type III was immunostained together with myelin basic protein (MBP) and SOX10, to evaluate the interaction between the regenerative SCs with the regrowing axons (Fig. 11A). The results showed that although NRG1 delivery increased the number of SOX10-positive Schwann cells, the expression of axonal NRG1 was significantly lower in the empty (MFI 262±39.55) and NRG1 groups (MFI 309.2±96.34) than in the autograft group (MFI 1231±122.8). The expression of MBP was consistent with that of axonal NRG1. Given the fact that axonal NRG1 type III is required for Schwann cell myelination ^12,32^, these results suggested that the lack of axonal NRG1 type III in the regenerated axons limited the axon remyelination in a 40 mm nerve gap injury.

## Discussion

**Figure 12.**
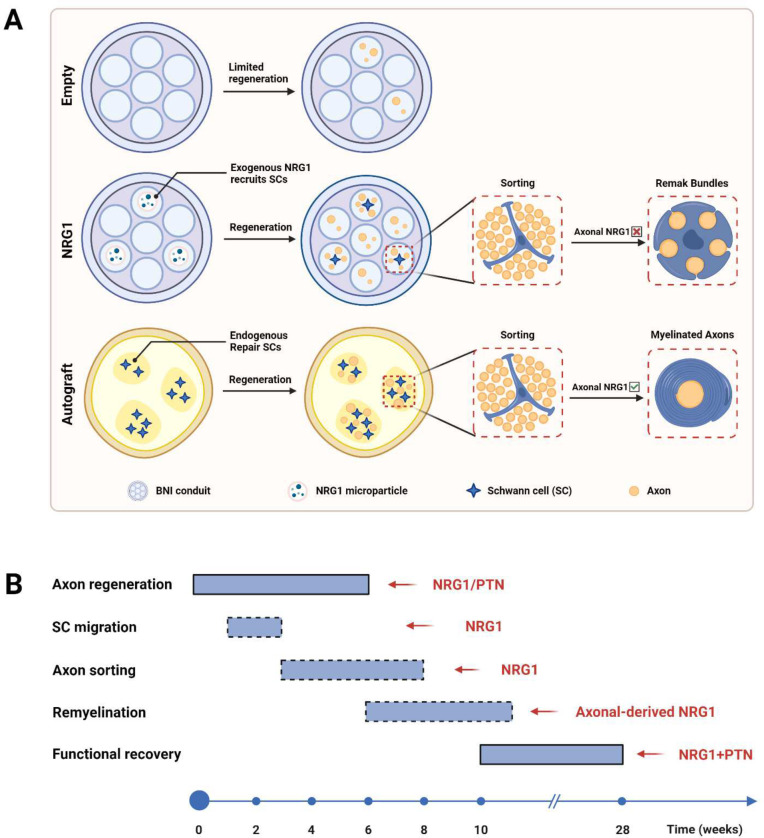
Schematic of cellular and molecular mechanisms involved in autograft and BNI critical gap repair. **A,** Axon sorting and remyelination outcomes resulted from different treatment strategies. In an empty BNI conduit, the number of Schwann cells in the middle of the conduit was limited and axon regeneration mostly fails. NRG1 delivery recruits pro-regenerative SCs to migrate and proliferate in the conduit. However, due to a lack of axonal NRG1 type III expression, remyelination of the regenerated axons is limited. In an autograft treated animal, axon regeneration and remyelination was well supported by the repair Schwann cells. **B**, Timing of critical steps involved in nerve regeneration and the molecules regulating these steps. Dashed boxes are used to indicate the putative timing of the event.

Growth factors are promising therapeutic proteins to be used in nerve conduits due to their critical role during nervous system development and elevated expression after nerve injury. Neuregulin 1 type III, a NRG1 isoform expressed by axons, has been implicated in developmental myelination via instructing Schwann cell proliferation and differentiation ^33,34^. The expression level of NRG1-III, which is proportional to the axon diameter, determines the ensheathment fate of axons ^12,32^. Here, we showed that slow NRG1 release in BNI conduits promotes axon sorting, remyelination, and some functional recovery across a 40 mm critical gap. Additionally, we combined NRG1 with PTN, a neurotrophic factor showing promising pro-regenerative effects in our previous studies ^9,29^, and confirm that the combination of pro-regenerative and pro-remyelinating facts further improves nerve regeneration and functional recovery. The proposed mechanisms underlying the improved nerve regeneration outcome resulted from NRG1 and PTN delivery are summarized in Fig. 12 and discussed in detail as follows.

Recovery of motor and sensory function after a nerve gap injury depends on successful and timely axon regeneration, axon sorting, remyelination, and reinnervation. Previous studies have revealed some of the signaling pathways involved in the regulation of these steps ^10,35,36^. In this study, we found the exogenously delivered NRG1 type III improved axon regeneration, as evidenced by the enhanced axon growth in the DRG culture assay and the increased axon number in the implanted nerve conduits. This is consistent with previous reports that genetic ablation of NRG1 resulted in a slower rate of axon regeneration in adult animals and the enforced expression its receptor ErbB2 on Schwann cells increased the number and diameter of regenerated axons ^34,37^. Similarly, some other growth factors have also shown a pro-regenerative effect, such as GDNF, NT-3, IGF-1, and FGF-2, etc ^38^. These findings collectively support the use of exogenously delivered growth factors to promote axon regeneration in nerve gap injuries.

Schwann cell migration is crucial to successful axon regeneration and remyelination in nerve gap injuries ^39^. NRG1 type III has been implicated in the regulation of Schwann cell migration during development in zebrafish and mice ^40,41^. In this study, we showed that the Schwann cell number in the nerve conduits were significantly increased by NRG1 type III treatment, providing *in vivo* evidence that NRG1 type III promotes Schwann cell migration during nerve regeneration. The activation of Schwann cell ErbB2 signaling by NRG1 could also explain the enhanced Schwann cell migration in the implanted conduits, given the fact that ErbB2 is the principal NRG1 receptor in Schwann cells and it directly activates the exchange factor Dock7 to promote Schwann cell migration ^42,43^. Signaling pathways downstream of NRG1/ErbB2 may include Src-FAK and PI3K-Akt-mTOR ^44,45^. However, NRG1 type III might not be essential to the migration of Schwann cells, as genetic knock-out of this molecule impaired but not completely inhibited the migration of Schwann cell precursors ^41^. Since additional signaling molecules have been identified as the regulator of Schwann cell migration, such as adhesion molecules and miRNAs, it remains to be determined which ones are crucial and whether the combination of these signaling molecules could further promote the migration of Schwann cells after nerve injury ^46–52^.

Radial sorting is a prerequisite for remyelination of regenerated axons. During development, radial sorting of axons is a multistep morphogenetic process, including formation of axon bundles surrounded by Schwann cells and a common basal lamina, insertion of Schwann cell processes into axonal bundles, recognition and radial segregation of large caliber axons, proliferation of Schwann cells to match the axon number, and finally defasciculation and establishment of 1:1 myelinated fibers or the formation of Remak bundles containing only small caliber axons ^10^. The molecular control of axon sorting is not fully understood. External signals that are known to trigger the radial sorting process include ECM components in the basal lamina such as laminin and collagen, axonal signaling molecules such as Wnt, as well as mechanical stimuli ^53–56^. NRG1 type III has been strongly suggested as one of the axonal signaling molecules that triggers radial sorting, since it is critically involved during developmental myelination ^12^. To examine whether exogenously delivered NRG1 III promotes axon sorting and subsequent remyelination, we performed TEM analysis to visualize the Schwan cell morphology in the nerve samples. As expected, NRG1 treatment improved Schwann cell sorting of unmyelinated axons, indicated by the reduced number of abnormal axon bundles. This provides evidence that NRG1 type III serves as an axonal signaling molecule that controls the radial sorting process during nerve regeneration.

In addition to promoting axon sorting, we expected NRG1 type III treatment would also promote remyelination. Surprisingly, NRG1 treatment did not increase the number of remyelinated axons versus the empty control, although it did improve the g-ratio. Given the critical role of membrane-bound NRG1 type III in myelin formation, the limited remyelination we observed could be explained by a lack of neuronal expression of NRG1 type III in the regenerated axons, as indicated by the negative staining results shown in Fig. 11. In line with this, it was found in a facial nerve transection model neuronal NRG1 type III level was significantly downregulated and did not recover until 14 days postinjury ^57^. Furthermore, genetic upregulation of NRG1 type III level in neurons and pharmacological suppression of its inhibitor tumor necrosis factor-alpha-converting enzyme (TACE/ADAM17) improved peripheral nerve myelination in mouse models of hypomyelinating neuropathies ^58,59^. Interestingly, the addition of PTN seems to have positive effects on remyelination, as indicated by the further reduced value of g-ratio, which implies the interdependence between axons and Schwann cells during nerve regeneration ^60^. Additional experiments are needed to further elucidate the influence of different promoting and limiting factors on remyelination after nerve injury.

Denervation muscle atrophy increases progressively following a nerve injury, characterized by reduced muscle mass, decreased cross-sectional area, increased connective tissue, and altered muscle fiber distribution ^61^. We found muscle mass and fiber area of all BNI groups were significantly lower than that of the autografts. The growth factor treatment did not significantly improve muscle mass and fiber area, implying the muscle recovery process during nerve regeneration is regulated by different signaling events ^62,63^. Notably, muscle mass in the autograft treated animals was significantly smaller than that of healthy animals, suggesting the presence of severe muscle atrophy despite the excellent axon regeneration and remyelination in these animals. Hence, by the time the regenerated axons reaching the target muscle, the denervated muscle fibers could already be too weak to reform neuromuscular connections.

Despite these mixed results, the PTN-NRG1 treated animals showed significant functional recovery compared with the empty BNI treated ones. Conversely, the TSI value of animals in the empty group did not show an upward trend as observed in PTN-NRG1 group throughout the recovery period. This suggests that although the empty group had detectable number of myelinated and unmyelinated axons, these were not enough to achieve functional recovery. It is interesting that NRG1 alone did not improve functional recovery significantly, but the combined treatment did, although we did not observe a significant increase of remyelinated axon number in the PTN-NRG1 group. NRG1 improved axon sorting, which might provide positive effects on nerve conduction by properly ensheathing and segregating regenerated axons. The greater number and diameter of unmyelinated axons in PTN-NRG1 group could also contribute to the improvement in toe-spread ^64^. In future experiments, histological labelling of muscle tissue will provide additional insight into how re-innervation is modulated at the neuro-muscular junction, and whether there are differential effects on sensory versus motor recovery.

In conclusion, this study provides evidence that exogenously delivered NRG1 promoted axon sorting and remyelination, and the combination of PTN-NRG1 further enhanced axon regeneration and functional recovery across a 40 mm critical nerve gap. Our results encourage further investigation and application of growth factors as therapeutic proteins for peripheral nerve gap injury treatment.

## Methods

### Microparticle fabrication and characterization

Poly(lactic-co-glycolic acid) (PLGA) microparticles encapsulating pleiotrophin (PTN) and neuregulin 1 type III (NRG1 type III) were fabricated using a previously described double emulsion method ^65^. Briefly, 20μg of NRG1 type III (R&D Biosystems) and 20μg of PTN (Invitrogen) were dissolved in deionized water separately as the inner aqueous phase. PLGA dissolved in dichloromethane (DCM) was the organic phase. The above two phases were sonicated to form the first emulsion, then dispersed in 1% poly(vinyl alcohol) (PVA) aqueous solution to form the second emulsion. DCM was evaporated from the solution by magnetic stirring in a fume hood over the course of four hours. Microparticles were collected by centrifugation at 5000 rpm for 15 minutes, frozen at −20°C overnight followed by lyophilization. A zeta potential analyzer was used to measure the size distribution of the resulting microspheres. As a no growth factor control, PLGA microparticles loaded with deionized water were prepared using the same procedure. Growth factor release from microparticles was determined by suspending microparticles in PBS at 37°C and collecting samples at different time points over one month for ELISA assay.

### Bioactivity of encapsulated protein

To assess whether the encapsulated growth factors were biologically active, dorsal root ganglia (DRG) explants were incubated with the microspheres and neurite growth was quantified. Briefly, neonatal mice (P0-P4) were used to harvest dorsal root ganglia explants. After cleaning the connective tissue, DRGs were placed on poly(D-lysine) (PDL) and laminin coated glass bottom wells and stabilized in place using 15μl collagen (EMD Millipore#ECM675). Microspheres mixed with collagen solution were placed in each well and incubated at 37°C for 15 minutes to allow the collagen to polymerize. Thereafter, 400μl neurobasal A media supplemented with 2% B27, 0.5% penicillin/streptomycin, and 0.75% L-glutamine was added to each well and incubated at 37°C, 5% CO_2_ for 7 days. Every 72 hours, 200μl of the media was replaced from each well with fresh media. After 7 days, the DRG explants were fixed using 400μl of 4% paraformaldehyde for 15 minutes on a shaker and rinsed with PBS. After rinsing, the explants were permeabilized using 0.5% Triton X100 in PBS and blocked with normal goat serum for one hour. The DRGs were then incubated with mouse anti-β-III tubulin overnight at 4°C. The next day, the explants were rinsed three times in PBS and then labelled with goat anti-mouse secondary antibody for one hour and counterstained with DAPI to visualize the cell nuclei.

### Surgical procedures

After confirming the sustained delivery and biological activity of the microspheres, a cohort of twenty-four adult female New Zealand white rabbits were implanted with biosynthetic nerve implants (BNI) as described previously ^9^. Briefly, the peroneal nerve of the rabbit was exposed through a 6 cm muscle-sparing incision in the biceps femoris muscle. The nerve was isolated from underlying connective tissue and a 30 mm length was transected and replaced by the BNI conduit filled with either Control (n=6), NRG1 (n=6) or PTN-NRG1 (n=6) microspheres. As a positive control (n=5), the resected nerve section was reversed and sutured to the nerve stumps by adding a 10 mm empty tube to compensate for nerve retraction, at the distal end. The BNI conduit consists of a transparent polyurethane tube (4 cm in length, 3 mm OD, 1.7 mm ID, Braintree Scientific Inc.) with 8 microchannels (7 of 0.4 mm and 1 in the center of 0.5 mm in diameter) filled with collagen-microsphere mixture, as described previously ^9^. In this study, the 4 cm BNI conduits were loaded with 3.6μl of microparticle suspension to release 2.16 ng/μl/day NRG1 type III estimated by ELISA. The study was double blinded and randomized, and all animal experiments were performed in accordance with the guidelines of the Institutional Animal Care and Use Committee of the University of Texas at Dallas, according to the NIH guide for the care and use of laboratory animals.

### Toe-spread index (TSI) assessment

The toe-spread index is the standard behavioral assessment used to evaluate the re-innervation of the peroneal nerve muscle group and is an indicator of functional recovery. When the animals are held loosely at the scruff of the neck and lowered suddenly, they extend their toes as a reflex to land. Videos were taken for each animal before surgery as baseline and weekly starting 5 weeks post-surgery to assess functional recovery. For each time point, the toe-spread between the injured and the un-injured foot was compared. The ImageJ software was used to measure the distance between the first and the last toe of the hind paw. The TSI value is calculated as the ratio of the injured over the uninjured toe-spread.

### Electrophysiological and muscle force measurements

In addition to recording compound motor action potentials (CMAPs) and compound nerve action potentials (CNAPs), measurement of muscle force is known to be a good indicator of functional motor recovery and successful re-innervation. Animals were sedated with ketamine (35mg/kg) and Xylazine (5mg/kg) combined in a single syringe and administered intraperitoneally. After that, the surgical plane of anesthesia was maintained using 2% isoflurane in oxygen. In a sterile field, the muscle was re-opened using a scalpel and the repaired nerve was re-exposed. After carefully isolating from surrounding tissue, two pairs of hook electrodes were placed proximal and distal (at 6 cm between them) to the repair site for stimulation and recording respectively. Using a sterile scalpel, the group of muscles innervated by the peroneal nerve was exposed and a pair of needle electrodes was placed for recording CMAPs. A nylon suture was connected to the tendon protruding at the ankle for attachment to a force transducer (RB-Phi-117, RobotShop, Mirabel, QC) to record isometric force from the muscles ^66^. The proximal end of the repaired peroneal nerve was stimulated to obtain CNAPs and CMAPs using the OmniPlex Neural Data acquisition system whereas the corresponding force exerted by the muscle was recorded using a custom interface programmed in MATLAB.

### Animal perfusion and tissue preparation

At the end of twenty-eight weeks, anesthetized animals were perfused with 4% paraformaldehyde. The regenerated nerve, with the implant tube intact, was harvested along with the respective *tibialis anterior* muscle. The tissue was then post-fixed in 4% PFA for 24–48 hours and then transferred to PBS solution. The regenerated nerve tissue was dissected, and the tube was separated carefully. Gross tissue analysis was performed to determine the extent of regeneration across the injury gap. The tissue was divided into six sections, proximal (P), middle (M1& M2) and distal (D1, D2 & D3), and processed for histological analysis.

### Immunohistochemistry

The harvested nerve tissue sections labelled P, M1, D1 and D3 were embedded in paraffin and 10 μm thin transverse sections were obtained using a microtome (Leica RM 2235). A heat induced antigen retrieval method was used to recover the epitopes. The tissue sections were then labelled with primary antibodies against different axon and Schwann cell markers, including mouse anti-β-tubulin III (1:400, Invitrogen), goat anti-NRG1 isoform SMDF (1:200, R&D Systems), chicken anti-MBP (1:400, Novus Biologicals), and mouse anti-SOX10 (1:400, Proteintech). After overnight incubation at 4°C, the sections were rinsed and then labelled with Cy2-, Cy3- and Cy5-conjugated secondary antibodies for two hours (1:200, Jackson ImmunoResearch). After rinsing, mounting media (Shandon^™^ Immu-Mount) was used to cover-slip the slides. Images were taken using a Nikon A1R confocal microscope. Fluorescence images were quantified using ImageJ.

### Transmission electron microscopy

Tissue sections M2 and D3 were post-fixed in 2.5% glutaraldehyde in 0.1 M cacodylate buffer and embedded in epoxy resin. 1μm sections were obtained and imaged using a JEOL 1400X transmission electron microscope. Six regions per animal were selected for quantification of axon number, axon diameter and g-ratio, using the ImageJ software. Axon diameter was calculated as the average of two diameter measurements at 90° from each other.

### Statistical analysis

All the graphs, calculations, and statistical analyses were performed using GraphPad Prism software version 9.0 for Mac (GraphPad Software, San Diego, CA, USA). The comparison of means between different groups of numerical variables was performed using one-way ANOVA. Homogeneity of variances was tested using Brown-Forsythe and Bartlett’s tests, and in a case of unequal SDs Brown-Forsythe and Welch ANOVA test was applied. If data were not normally distributed, the comparison of medians between different groups was switched to non-parametric one-way ANOVA. P value less than 0.05 was considered statistically significant. Significance level: *, p<0.05; **, p<0.01; ***, p<0.001; ****, p<0.0001; ns, p>0.05. Error bars in the graphs represent standard deviation.

## Figures and Tables

**Figure 1.**
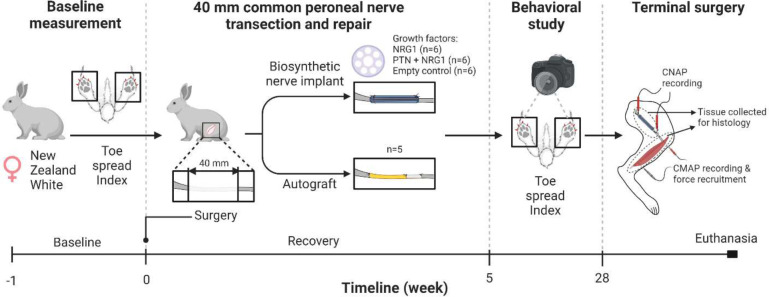
Experimental design. Adult female New Zealand White rabbits underwent baseline toe-spread testing. A 40 mm common peroneal nerve gap injury was repaired using BNIs with sustained release of either NRG1, PTN, or PTN+NRG1. Toe-spread index was evaluated weekly starting at week 5 and until week 28, time at which the nerves were re-exposed for muscle and nerve physiology, followed by euthanasia and tissue harvesting.

## Data Availability

All data needed to evaluate or reproduce the conclusions in the paper are present in the paper. The datasets used and/or analyzed during the current study available from the corresponding author on reasonable request.
